# Case Series of Autoimmune Pancreatitis and IgG4-Related Sclerosing Cholangitis

**DOI:** 10.7759/cureus.26657

**Published:** 2022-07-08

**Authors:** Anass Nadi, Yassamin Benhayoun, Reda Cherkaoui, Hanane Delsa, Fedoua Rouibaa

**Affiliations:** 1 Gastroenterology and Hepatology, Faculty of Medicine, Mohammed VI University of Health Sciences (UM6SS), Casablanca, MAR; 2 Gastroenterology, Faculty of Medicine, Mohammed VI University of Health Sciences (UM6SS), Casablanca, MAR; 3 Radiology, Faculty of Medicine, Mohammed VI University of Health Sciences (UM6SS), Casablanca, MAR

**Keywords:** diagnosis criteria, corticosteroids, auto-immune pancreatitis, igg4-relatedcholangitis, igg4-related disease

## Abstract

IgG4-related disease (IgG4-RD) is an emerging immune-mediated disease that can involve any organ. The involvement of the pancreas and biliary tract is the most common and well-studied in the literature. It is characterized by a non-specific presentation, mimicking a malignant process. The goal was to look at the different clinical and paraclinical aspects of this disease, as well as the challenges that come from its management. It was made up of three observations of patients with IgG4-RD involving the biliary tract and pancreas. The first observation concerned intrahepatic biliary cholangitis that was accompanied by porto-mesenteric thrombosis, which was discovered by cholestatic jaundice on the 15th day after an appendectomy, and the patient improved under corticosteroids and anticoagulants. The second observation concerned an acute revelation of the disease. It was an acute attack of chronic pancreatitis of IgG4-RD. The main symptoms were pancreatic pain and exocrine pancreatic insufficiency, and corticosteroid therapy allowed remission. The third observation was related to autoimmune pancreatitis (AIP) and IgG4-related sclerosing cholangitis, revealed by jaundice with cholestasis. The patient acquired corticosteroid resistance and an adverse progression to decompensated cirrhosis, and liver transplantation was indicated. The clinical presentation of IgG4-RD is heterogeneous, as evidenced by our three clinical observations. There are still significant gaps in our understanding, particularly in terms of pathogenesis and factors that influence therapy response. Further observational and interventional research is needed to better manage this disease.

## Introduction

The discovery of IgG4-related disease (IgG4-RD) has revolutionized pancreatic and biliary autoimmune disease management. It is a recently discovered disease characterized by fibro-inflammatory attacks that can affect one or several organs in a synchronous or metachronous way [1]. The epidemiology of this condition is poorly characterized in the literature, with an estimated annual incidence of 0.28-1.08 per 100,000 people [2]. The diagnosis can be difficult to make, but it is histologically verified. The presence of lymphoplasmacytic infiltration, irregular storiform fibrosis, and obliterative phlebitis is the main criteria. Several worldwide consensuses have validated other specific criteria.

The pancreatic localization of igG4 disease was the first to be described, and thus the most investigated. It usually manifests as a chronic pseudotumor; however, acute manifestations have been described. IgG4-RD can be mistaken for a variety of conditions, including pancreatic adenocarcinoma.

Although biliary tract involvement is most often associated with pancreatic involvement, isolated sclerosing cholangitis has been observed in certain cases. It shares certain similarities with cholangiocarcinoma. IgG4-RD's biliopancreatic involvement is known to be steroid-sensitive with a significant risk of relapse or cortico-resistance. Immunomodulatory therapies such as thiopurines, methotrexate, and rituximab have been shown to be necessary in some cases [3].

The purpose of this case series is to focus on the current status of biliary and pancreatic IgG4-RD, as well as the challenges in diagnosing and managing it. We also want to remind specialists that they should rule out pancreatic or biliary cancers first to avoid unnecessary surgery for a steroid-sensitive disease.

## Case presentation

Case 1

A 60-year-old male patient was admitted to our gastroenterology unit on the 15th day of an appendectomy. He presented with jaundice for four months with pruritus, abdominal pain, and asthenia. His medical history only included transurethral resection of the prostate in 2021.

On examination, the patient presented with generalized mucocutaneous jaundice with scratch marks. An abdominal examination showed tenderness in the right iliac fossa. Laboratory tests showed leukocytosis and an elevated C-reactive protein, cholestasis with unconjugated hyperbilirubinemia, and without cytolysis (Table 1).

**Table 1:** Laboratory findings for the patient in case 1 ALP: alkaline phosphatase; GGT: gamma-glutamyltransferase; AST: aspartate transaminase; ALT: alanine transaminase; WBC: white blood cells; CRP: C-reactive protein

Labs	Values at admission	Values at day 30 of corticosteroids	Values at six months after stopping corticosteroids
ALP (U/L)	1500	109	98
GGT (U/L)	600	78	63
AST (U/L)	42	38	35
ALT(U/L)	39	36	32
Bilirubin (mg/dl)	35	12.5	10.5
WBC (mm³)	14160	8500	9000
CRP (mg/l)	174	6	8

**Figure 1:**
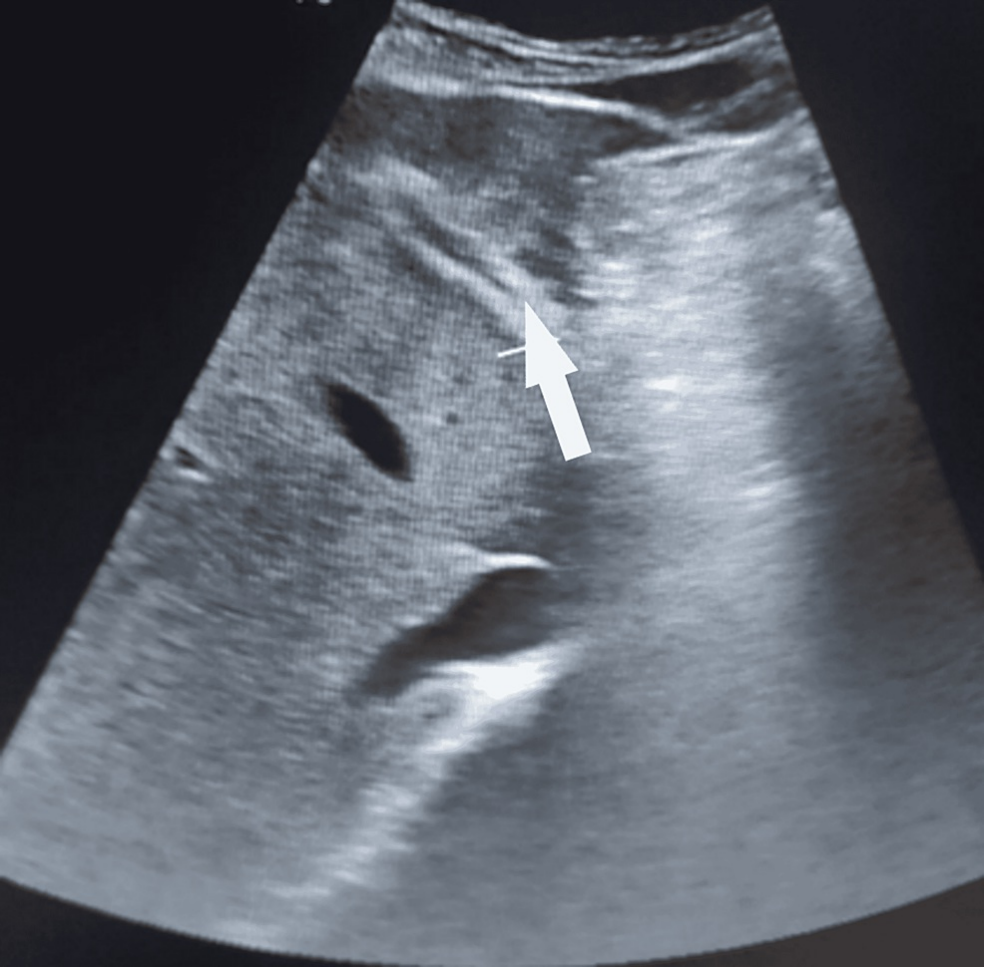
Abdominal ultrasound demonstrating thickening and intrahepatic bile ducts (white arrows)

An abdominal ultrasound showed thickening and intrahepatic bile duct (IHBD) dilatation in the left hepatic lobe, a portal and mesenteric thrombosis, as well as an abscess in the right iliac fossa. The gallbladder and the main bile duct were normal (Figure 1).

**Figure 2:**
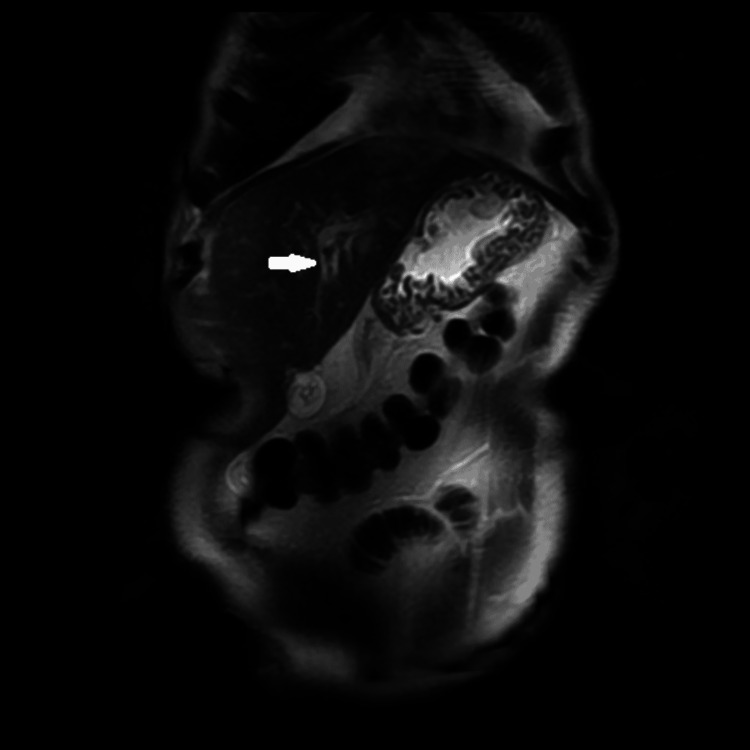
Magnetic resonance cholangiopancreatography showing thickening and intrahepatic bile ducts in left liver lobe (white arrows)

Magnetic resonance cholangiopancreatography (MRCP) confirmed the dilatation of the IHBD of the left liver lobe with regular wall thickening without any visible obstruction (Figure 2).

The patient underwent ultrasound-guided drainage for the abscess in the right iliac fossa, and then he received antibiotics and anticoagulants for the porto-mesenteric thrombosis. For cholestasis, the initial etiological assessment was negative, including hepatitis B and C virus serology, and autoimmune tests including antinuclear antibodies (ANA), anti-native DNA antibodies (anti-DNA), anti-smooth muscle antibodies (ASMA), anti-mitochondrial antibodies (AMA), anti-soluble liver antigen autoantibodies (SLA), and anti-liver-kidney microsomal antibody (anti-LKM). The biopsy of bile duct thickening showed non-specific lymphoplasmacytic inflammatory infiltrates with no signs of malignancy, while the serum level of IgG4 was elevated to 1.820 g/l (0.040-0.870g/l), carbohydrate antigen19-9 was slightly elevated at 55 U/ml (˂37 U/mL).

Ultimately, the diagnosis of IgG4-related sclerosing intrahepatic cholangitis was withheld. Corticosteroid was started from 0.6 mg/kg/day, which is equivalent to 50 mg of prednisolone daily for one month, tapered gradually (a decrease of 5 mg every two weeks). At present, after six months of withdrawing the corticosteroids, the patient is asymptomatic with complete resolution of jaundice, normalization of the liver biochemical tests, and a normal IgG4 serum level. The follow-up abdominal Doppler ultrasound showed a normal liver without any dilatation or thickening of the IHBD with complete re-permeabilization of the portal veins and superior mesenteric veins. A regular follow-up will be performed every six months by clinical examination, abdominal ultrasound, liver biochemical tests, and IgG4 serum level.

Case 2

A 35-year-old male male patient was referred to the emergency department for acute epigastric pain with diarrhea. His medical history includes recurrent acute pancreatitis in 2015 and 2020. On the physical exam, there was epigastric tenderness. Lipasemia was elevated to 190 U/l, the liver biochemical tests were normal, the lipidic tests showed slight hypercholesterolemia at 2.2 g/l, and the phosphocalcic test was normal. The fecal elastase was low at 109 μg/g of stool (normal above 200μg/g of stool).

We first suspected an acute flare of chronic pancreatitis. The autoimmune origin was the most probable, given the absence of alcoholism, biliary stones, and metabolic abnormalities. The Ig4 serum level was high at 1.71 g/l (0.040-0.870 g/l), so we withheld the diagnosis of autoimmune pancreatitis (AIP) type 1 with exocrine pancreatic insufficiency. A biopsy was not performed because of the normality of the imaging (abdominal CT and MRCP and echo-endoscopy). Corticosteroid therapy was started with 40 mg of prednisolone for one month, then progressive degression was started. The patient is on the 12th week of corticosteroids with the disappearance of epigastric pain and signs of pancreatic insufficiency. The IgG4 serum level was normal.

Case 3

A 63-year-old male patient was referred to our department for cholestatic jaundice evolving for one month with chronic diarrhea associated with asthenia and weight loss. He had no known past medical history. The clinical examination revealed generalized jaundice with right upper quadrant tenderness.

**Figure 3:**
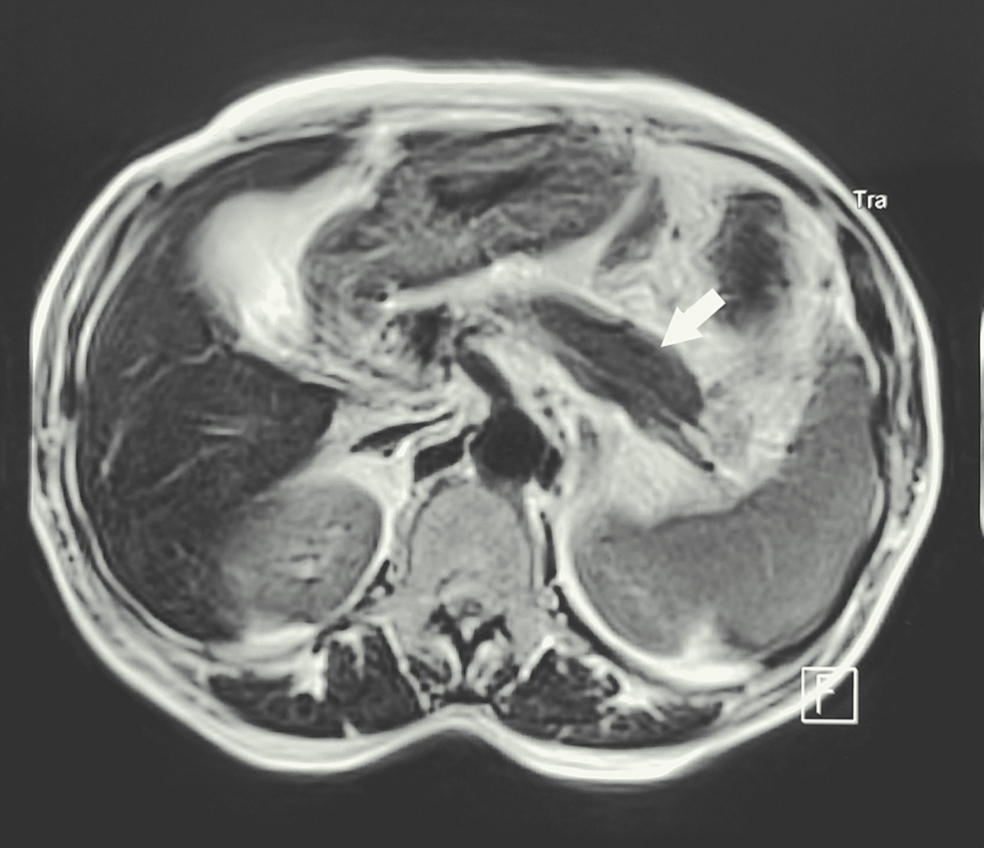
Magnetic resonance cholangiopancreatography showing enlarged pancreas

Initial labs showed cholestasis with elevated bilirubin (40.4 mg/L), conjugated bilirubin (39 mg/l), GGT (640U/l), ALP (787U/l), and cytolysis (AST: 77U/l, ALAT: 140U/l). Abdominal CT showed a body enlarged pancreas. MRCP revealed a normal common bile duct and dilated intrahepatic ducts with an enlarged pancreas (Figure 3). Echo-endoscopy showed the same findings.

Serology for viral hepatitis and autoimmune laboratory testing came back negative. The serum level of IgG4 was raised to 2.04 g/l. Functional stool investigations showed elevated total fat at 27.65 g/24 h (2 to 6 g/24 h), alpha-1 antitrypsin was normal, and fecal elastase was less than 50 μg/g of stool. The patient underwent a liver biopsy, which showed extensive portal chronic inflammation with ductular proliferation. The portal inflammation was composed mostly of lymphocytes with focal scattered plasma cells and a few eosinophils. The bile ducts showed focal injuries with ductular proliferation, prominent interface hepatitis, and scattered lobular inflammatory infiltrates with hepatocyte damage were also noted.

We finally retained the diagnosis of IgG4 sclerosing cholangitis with autoimmune pancreatitis. Corticosteroid therapy at 0.6 mg/kg/day of prednisolone was started for four weeks, then tapered to 5 mg/two weeks. After three months of corticosteroid therapy, jaundice disappeared, but the patient kept asthenia with cytolysis and cholestasis. The decision was to add azathioprine at a dose of 2 mg/kg/day and to maintain corticosteroid therapy at a dose of 5 mg/day. After three months of azathioprine, the patient developed cirrhosis with ascitic decompensation. We decided to stop corticosteroids and azathioprine and discussed liver transplantation.

## Discussion

IgG4 disease is a recently discovered immune-mediated condition affecting several organ systems. The characteristics of this entity have been the subject of an international consensus adopted in 2012 [4], and several diagnostic criteria have been established, from the Japanese Comprehensive Diagnostic Criteria (CDC) [5] to the ACR/EULAR classification criteria in 2019 [6]. Other organ-specific criteria have been proposed.

Its epidemiology is still poorly described. In Japan, the prevalence is estimated at 1/600,000 and the annual incidence at 0.28-1.08/100,000 inhabitants [2]. Its pathophysiology has been progressively revealed thanks to the multiplication of publications in recent years. In our series, we reported three cases illustrating the most frequent and problematic localizations of IgG4-RD, namely pancreatic and biliary involvement.

The pancreatic involvement was the first described manifestation. It causes an enlarged pancreas with progressive parenchymal destruction due to the extension of fibrosis [4]. The average age of diagnosis is 60 years, with a male predominance [7]. It accounts for 96% of AIP cases in Japan and at least 80% in the West. Pancreatic involvement constitutes 41% of the locations of IgG4-RD [8]. In an American series, 2.5% of duodenopancreatectomy performed for suspected cancer was found to be AIP [9]. The dilemma of cancer simulation has prompted international groups to develop a set of diagnostic criteria that incorporate clinical, radiological, histological, biological, and therapeutic responses. In our series, we used the International Consensus Diagnostic Criteria (ICDC) to establish the diagnosis of IgG4 pancreatitis [10]. The clinical presentation is often progressive, rarely acute, with jaundice in 75% of cases and pruritus, steatorrhea, and diabetes indicating exocrine and endocrine pancreatic insufficiency in more than 60% of cases [8]. On radiology, an enlarged pancreas with a lack of lobulation and stenosis of the pancreatic ducts is seen. The enlargement can sometimes be localized, raising the possibility of cancer. Echo-endoscopy can reveal comparable anomalies and allow pancreatic biopsies [10].

Histology is the gold standard for diagnosis. Characteristic pathologic features are lymphoplasmacytic infiltrates with prominent IgG4+ plasma cells, storiform fibrosis, and obliterative phlebitis. In practice, it is difficult to obtain a representative pancreatic sample with fine needles by transparietal approach or guided by echoendoscopy.

The IgG4 serum level is of major interest in establishing the diagnosis; a threshold of 135 mg/dl has been retained for the positive diagnosis because it distinguishes between pancreatic cancers and chronic calcifying pancreatitis; this threshold has a sensitivity of 90% and a negative predictive value of 96%, but the specificity and positive predictive value are low at 60% and 34%, respectively [11].

In our series, we reported two cases of IgG4 pancreatitis with diverse clinical presentations. The first observation concerned acute recurrent pancreatitis with normal imaging, a high IgG4 level, and exocrine pancreatic insufficiency. Similar cases have been reported as single clinical cases [12]. We considered it an early stage of the disease and intend to conduct a radiological follow-up. The second observation described a typical case of auto-immune pancreatitis associated with IgG4-related cholangitis.

IgG4-related cholangitis is second only to pancreatic involvement in IgG4 disease, which is associated in 83% of cases. Several criteria have been established for the diagnosis of IgG4 cholangitis; the most widely used are the Japanese ones published in 2012 [13]. The prevalence is 10% according to an American series of 125 patients and 6% according to another Japanese series of 235 patients [14]. Two patients in our series had intrahepatic cholangitis, which was isolated in the first case and accompanied by IgG4 pancreatitis in the third.

IgG4 cholangitis is characterized mostly by jaundice, as well as cytolysis and cholestasis. Imaging frequently shows biliary tract stenosis with thickenings. It primarily affects the extrahepatic bile ducts. Isolated intrahepatic involvement is possible but uncommon. A radiological classification has been established, individualizing four forms based on the levels of biliary stenosis. These features are not unique to IgG4 cholangitis [15].

Elevated IgG4 levels are important but inconstant criteria for the diagnosis. It can be normal in 10% to 20% of cases of IgG4 cholangitis. A threshold of 135 mg/dl seems to be useful to distinguish IgG4 cholangitis from pancreatic cancer and primary sclerosing cholangitis (PSC). However, a threshold of 182 mg/dl has been recommended with a specificity of 96.6% for distinguishing CS-IgG4 from cholangiocarcinoma [15]. A new quantitative PCR test that measures the IgG4/IgG RNA ratio has recently been proposed to differentiate IgG4 disease from pancreato-biliary malignancies. However, more research is needed to confirm this test on a larger scale [16]. All three of our patients showed elevated IgG4 levels considerably over the 135 mg/dl threshold, which was used to differentiate between diseases, most notably cancers.

In histopathology, IgG4 cholangitis presents the same common histological characteristics as IgG4-RD, including lymphoplasmacytic infiltrates with prominent IgG4+plasma cells, storiform fibrosis, and obliterative phlebitis. However, these criteria are difficult to demonstrate in the case of intrahepatic involvement [17].

When we did liver biopsies on two of our patients who had intrahepatic lesions on imaging, we discovered extensive lymphoplasmacytic infiltrates but no malignant cells. Because the immunohistochemistry study was inconclusive, we relied on other clinical, biological, radiological, and therapeutic responses to establish the diagnosis.

IgG4-RD is a corticosteroid-sensitive disorder with a 97% to 100% response rate. Western teams start with 0.6 to 0.8 mg/kg/day for four weeks, then reduce it by 5 mg every two weeks for three to six months. To limit the risk of relapse, most Japanese teams continue low-dose corticosteroids (2.5 to 5 mg/day) for three years [18]. Monitoring is clinical, radiological (by MRCP), and biochemical (by IgG4 serum level).

IgG4-RD is a recurring pathology, with a relapse incidence ranging from 26% to 70% after stopping corticosteroids [18]. Relapse risk factors include an initially high level of IgG4, a delayed decrease in IgG4 under corticosteroids, diffuse pancreatic involvement, jaundice, type II, III, or IV cholangitis, and disease involvement of more than two organs.

In case of relapse, immunosuppressive drugs are recommended along with corticosteroids. Azathioprine is the most commonly used, although the level of evidence is low. Rituximab is a promising treatment for the management of relapse and maintenance of remission [3].

In our case series, two patients have responded well to corticosteroid therapy without relapse to date, but the third patient has developed decompensated cirrhosis despite the addition of azathioprine.

## Conclusions

The IgG4-related disease is an emerging and poorly understood disease. Nearly two decades ago, it was identified as a distinct systemic disease entity. Pancreatic and biliary involvement are the most frequent involvement. They mainly mimic pancreatic cancer and cholangiocarcinoma. To avoid diagnostic errors, several diagnostic criteria have been validated by international consensus to avoid diagnostic errors. More prospective studies are needed to better define IgG4 illness and fill gaps in our understanding of pathophysiology and diagnosis. Furthermore, international consensus management of this disease in its early stages in the event of relapse is required. We hope our cases contribute to the literature pool. Ultimately, a complete understanding of the pathophysiology and evidence-based management guidelines awaits further studies.
